# Increased risk of perforated appendicitis in patients with schizophrenia and dementia: A population-based case-control study

**DOI:** 10.1097/MD.0000000000018919

**Published:** 2020-01-31

**Authors:** Huang Ren Lin, Hsiang Chi Wang, Jen Hung Wang, Hsin Han Lu

**Affiliations:** a Department of Family medicine, Lo-Hsu Medical Foundation, Lotung Poh-Ai Hospital, Yilan; b Graduate Institute of Clinical Medical Science, China Medical University, Taichung.; c Department of Medical Research, Hualien Tzu Chi Hospital, Buddhist Tzu Chi Medical Foundation; d Department of Family Medicine, Buddhist Tzu Chi General Hospital, Hualien, Taiwan.

**Keywords:** appendicitis, dementia, mental disorders, perforated appendicitis, schizophrenia

## Abstract

Previous studies have suggested that patients with psychotic or mental disorders are relatively pain insensitive, resulting in difficulties in the diagnosis of acute intra-abdominal diseases requiring emergency surgeries. We aimed to evaluate whether central nervous system (CNS) or mental disorders are associated with perforated appendicitis in patients with acute appendicitis.

We conducted a population-based case-control study using Taiwan's National Health Insurance Research database. Patients aged >18 years who had been hospitalized with a diagnosis of acute appendicitis between 2000 and 2013 were identified. After 1:1 matching for age and sex, 2792 patients with perforated appendicitis (case group) and 2792 patients with nonperforated appendicitis (control group) were included. CNS disorders, mental disorders, pain control medication, and several comorbidities were analyzed for the odds of appendiceal perforation with 95% confidence interval (CI) using the multivariable logistic regression model.

Schizophrenia and dementia were associated with a high risk of appendiceal rupture in patients with acute appendicitis, with an adjusted odds ratio of 2.01 for dementia (95% CI: 1.19–3.39, *P* = .009) and 4.8 for schizophrenia (95% CI: 1.62–14.19, *P* = .005). Other factors, such as other CNS disorders, comorbidities, and pain control medication, were not associated with the risk of perforated appendicitis.

Dementia and schizophrenia are associated with perforated appendicitis in patients with acute appendicitis. This might be owing to altered pain perception, difficult symptom expression, and delayed hospitalization. Further studies are still needed to determine the underlying mechanism and confirm the causality.

## 1 Introduction

Acute appendicitis is the most common abdominal surgical emergency in the world,^[1]^ and its lifetime risk is 12% in males and 23.1% in females.^[2]^ Usually, the treatment is based on whether the appendicitis is perforated. For example, nonoperative treatment is reasonable when ruptured appendicitis is complicated with peri-appendiceal abscess.^[3]^ Generally, the perforation rate in acute appendicitis is approximately 13% to 20%,^[4]^ and it may be owing to delayed presentation.^[5]^ Perforated appendicitis accounts for more mortality and morbidity than those without.^[6]^

Previous study reports indicate that patients with psychotic disorders such as schizophrenia are relatively pain insensitive which often leads to difficulties in the diagnosis of acute intra-abdominal diseases requiring emergency surgeries.^[7]^ In that study,^[7]^ the investigators reported their experience with 5 critical ill patients who had chronic schizophrenia and decreased pain perception; the lack of signs and symptoms in these patients resulted in delayed abdominal surgery. Moreover, pain insensitivity may also cause delay in the presentation of acute appendicitis, resulting in perforation.^[8]^ In a retrospective cohort study, it was observed that appendiceal perforation was more frequent in patients with schizophrenia than in controls.^[9]^ Another population-based retrospective study suggested that schizophrenia is associated with a 2.83 times increased risk of ruptured appendix in those who were hospitalized for appendicitis.^[10]^ In addition to psychiatric disorders, altered pain perception is also common in older patients owing to cognitive impairment (e.g., dementia), medication for chronic pain and multiple coexistent diseases.^[11]^ Hence, the precise diagnosis of acute appendicitis is also challenging in the elderly.^[12]^ Therefore, the incidence of perforated appendix is high in them.^[13,14]^ However, the association between the risk of perforated appendicitis and central nervous system (CNS) disorders has not been clearly reported. In light of existing evidence, we hypothesized that dementia and schizophrenia are associated with perforated appendicitis in patients with acute appendicitis. Here, we present data on a nationwide population-based case-control study that analyzed the association between ruptured appendicitis and mental or CNS disorders.

## 2 Materials and methods

### 2.1 Data sources

Taiwan launched its single-payer and mandatory-enrollment National Health Insurance (NHI) program in 1995. More than 99% of the Taiwanese are covered by the NHI program. All outpatient, inpatient and emergency services are covered by the NHI. This study was conducted by using Longitudinal Heath Insurance Database (LHID). The LHID, a subset of the Taiwan's NHI Research Database, contains data from 1 million people who were randomly sampled from the 23.8 million NHI beneficiaries for research purposes by the National Health Research Institute of Taiwan. The diagnostic codes used in LHID were from the International Classification of Diseases, 9th Revision, Clinical Modification (ICD-9-CM). To protect patient privacy and data security, personal identifiers in the LHID were encrypted before the National Health Research Institute released the database. This study was approved by the Research Ethics Committee of China Medical University and Hospital in Taiwan (CMUH-104-REC2-115). Informed consent was not required for this observational study according to our institutional guidelines.

### 2.2 Selection of case and control populations

The case and control populations were obtained from the LHID. Our study population included adult patients aged ≥18 years who were hospitalized (index admission) owing to acute appendicitis (ICD-9-CM code 540, 540.0, 540.1, or 540.9) between 2000 and 2013. The patients with perforated appendicitis (ICD-9-CM code: 540.0, 540.1) were classified as the perforated appendicitis group (case group) and the patients with nonperforated appendicitis (ICD-9-CM code: 540.x, except for 540.0, 540.1) were classified as the control group by 1:1 propensity score matching for age and sex.

### 2.3 Exposure assessment

We identified whether patients had been diagnosed with mental or CNS disorders before the hospitalization for acute appendicitis. Patients were categorized as having a mental or CNS disorder if there was at least 1 inpatient or 2 outpatient records as diagnosed by a neurologist, neurosurgeon, or psychiatrist before the index admission. CNS disorders included dementia (ICD-9-CM code 290.0–290.9, 294.1, and 331.0), Parkinsondisease (ICD-9-CM 332), cerebrovascular diseases (ICD-9-CM 430–438), and other CNS disorders (ICD-9-CM 330, 333, 340, 341–344, 348, 349). Mental disorders included schizophrenia (ICD-9-CM 295), affective disorder (ICD-9-CM 296), and other mental disorder (ICD-9-CM 291–294, 297, 300–302, 306, 310, 311, 315–319).

### 2.4 Covariates

Potential confounders such as diabetes (ICD-9-CM codes 250), hypertension (ICD-9-CM codes 401–405), ischemic heart disease (ICD-9-CM codes 410–414), chronic obstructive pulmonary disease (ICD-9-CM codes 490–496), chronic kidney disease (ICD-9-CM codes 580–589), cirrhosis (ICD-9-CM codes 580–589), and malignancy (ICD-9-CM codes 140–208, 209.0–209.3) were retrieved. Those comorbidities were defined as a disease diagnosed in at least 1 inpatient record or 2 outpatient records within 1 year before the index admission. Baseline medication for pain control was defined as a drug that had been prescribed within 3 months before the index admission, including non-steroidal anti-inflammatory drug (NSAID), opioids, and steroids by prescription code in National Health Insurance Research Database (NHIRD). Monthly income, urbanization, hospital geography, and hospital accreditation were also considered. Income was categorized into 3 levels (New Taiwan dollars ≥20000, 15000–19999, and <15,000) based on the income-related NHI premiums. Urbanization levels of residence were categorized into 4 levels (level 1 indicated the most urbanized areas and level 4 the least urbanized).

### 2.5 Statistical analysis

Continuous variables were compared using *t*-tests and categorical variables were compared by *χ*^2^ tests. The logistic regression model was used to calculate the adjusted odds ratio (OR) with 95% confidence interval (CI) for risks of perforated appendicitis associated with mental or CNS disorders. The multivariate logistic regression model was performed with adjustments for all potential confounding factors as listed in Table 1. All analyses were conducted using SAS 9.2 (SAS Institute Inc., Carey, North Carolina, USA). A two-sided *P* < .05 was considered statistically significant.

**Table 1:**
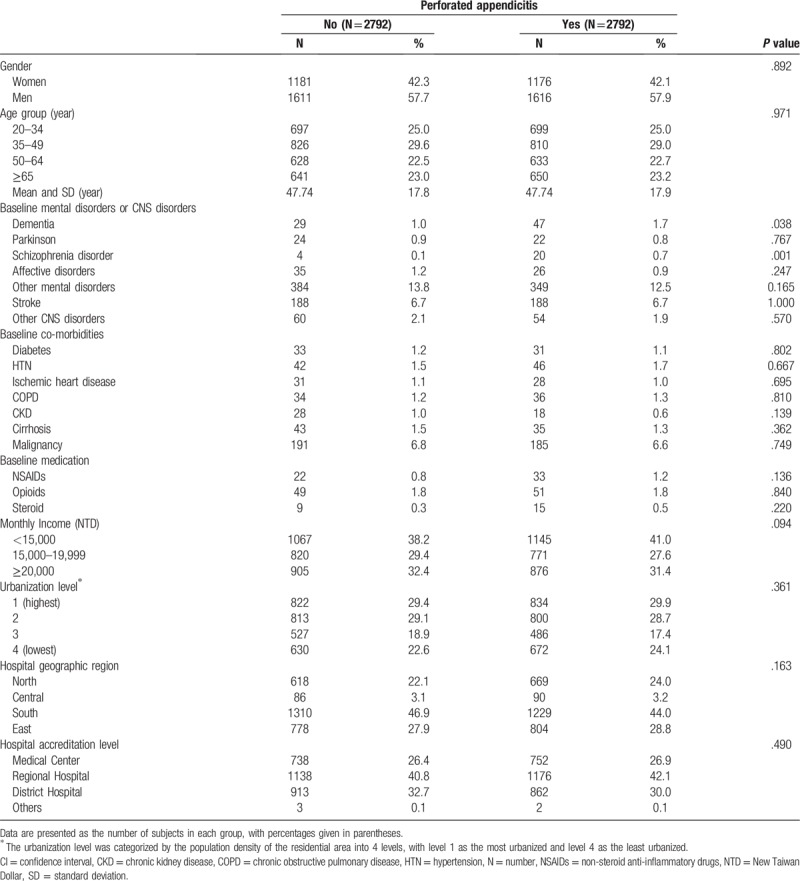
Baseline characteristics between perforated appendicitis group and non-perforated appendicitis group.

## 3 Results

Table 1 shows the patient characteristics of case and control groups. There were no obvious differences between the 2 groups in terms of distribution of age, sex, monthly income, urbanization level, hospital geographic region, and hospital accreditation level. There were significant differences in terms of schizophrenia disorder (0.7% vs 0.1%, *P* = .001) and dementia (1.7% vs 1.0%, *P* = .038) between the 2 groups.

Table 2 contains the adjusted OR of appendiceal rupture in patients with acute appendicitis for each variable listed in Table 1. Subjects with schizophrenia or dementia were associated with a high risk of appendiceal rupture, with an adjusted OR of 4.8 in schizophrenia (95% CI: 1.62–14.19, *P* = .005) and 2.01 in dementia (95% CI: 1.19–3.39, *P* = .009). However, subjects with other mental disorders were associated with a reduced risk of appendiceal rupture (adjusted OR = 0.78, 95% CI: 0.65–0.93, *P* = .005). Other factors, such as affective disorder, Parkinson disease, stroke, and other CNS disorders, were not associated with a risk of perforated appendicitis. All other baseline comorbidities and medication for pain control were not associated with an increased risk of developing perforated appendicitis.

**Table 2:**
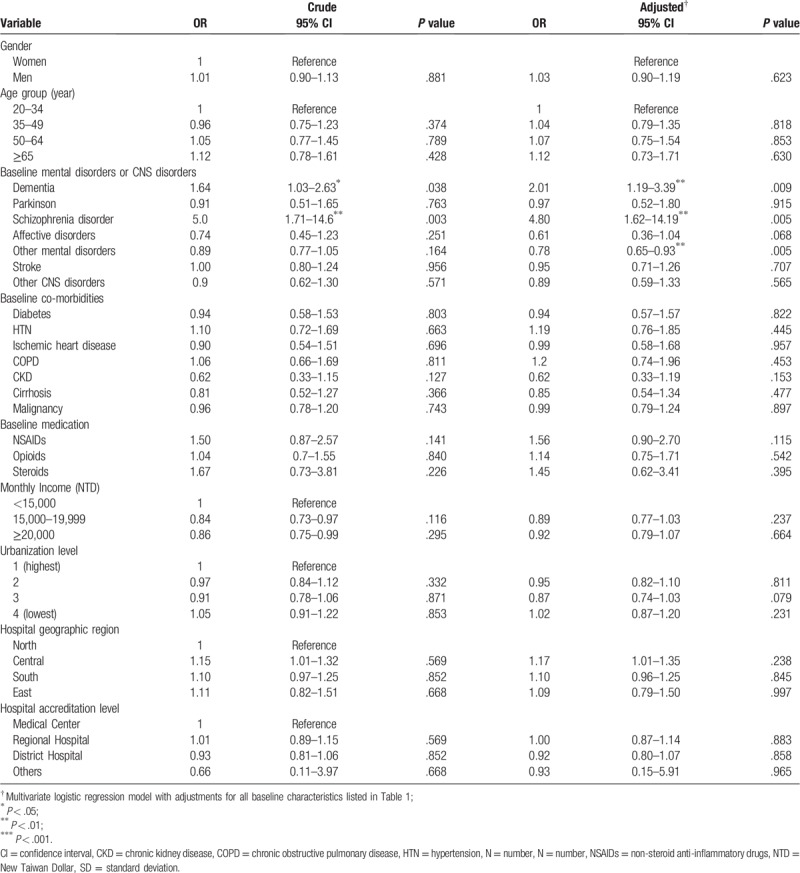
Odds ratios and 95% confidence intervals of perforated appendicitis associated with mental disorders and covariates.

## 4 Discussion

Data in this nationwide population-based case-control study indicate that schizophrenia and dementia were associated with an increased risk of perforated appendicitis among patients with acute appendicitis. Other factors, such as affective disorder, Parkinson disease, stroke, and other CNS disorders, were not associated with the risk of perforated appendicitis. In addition, all other baseline comorbidities and medicine usage for pain control were not associated with an increased risk of developing perforated appendicitis.

Previous studies have demonstrated the possible association between perforated appendicitis and schizophrenia.^[9,10]^ One retrospective cohort study included 62 patients with schizophrenia and 200 without schizophrenia requiring appendectomy (N = 1821). The adjusted OR for perforation was 4.87 (95% CI: 2.33–10.2) for schizophrenia, 2.18 (95% CI: 1.12–4.27) for delayed presentation and 3.35 (95% CI: 1.51–7.45) for age > 55 years.^[9]^ Another population-based retrospective study using NHI data in Taiwan also found that patients with schizophrenia have a 2.83 times higher risk of having ruptured appendicitis (adjusted OR = 2.83, 95% CI: 2.20–3.64). Age was also found to be a contributor to perforated appendicitis. However, in that study, there was no significant difference with respect to perforation risk in affective psychoses or other mental disorders compared with controls.^[10]^ However, there were no sufficient data for other factors influencing pain sensitivity or cognition function in these previous studies. Our study evaluated other related factors such as stroke, Parkinson disease, dementia, and other CNS disorder which can affect pain sensitivity or cognitive function. The use of medication for pain control was also analyzed and was not found to be associated with ruptured appendicitis.

Altered pain perception and high pain thresholds in patients with schizophrenia may result in delayed emergent operation for appendicitis, resulting in perforation.^[8,15,16]^ Our study revealed a 4.8 times higher perforation rate in the case group than that in the control group, which is similar to that reported by a previous retrospective study.^[9]^ In addition to schizophrenia, we found that dementia also has a significant association with perforated appendicitis (adjust OR = 2.01, 95% CI: 1.19–3.39), which may explain the increased risk of perforated appendicitis in the elderly reported by previous studies.^[13,14,17]^ One explanation for these data is that elderly patients, particularly those with cognitive dysfunction such as dementia, may have difficulty describing their symptoms which could mask the severity of the appendicitis. Pain threshold also increases with age^[18]^ which can result in delayed hospitalization and may also contribute to the high risk of perforation in acute appendicitis.^[11,19]^ In addition to ruptured appendix, previous investigators have reported several cases of schizophrenia patients presenting pain insensitivity of painful medical condition like peptic ulcer, peritonitis, acute abdomens, perforated bowel, and compartment syndrome, which resulted in tragic outcomes such as late surgical attention, misdiagnosis, and delayed treatment.^[20]^ Of note, in medical students and doctors, there are more stigmatizing attitudes toward schizophrenia patients about dangerousness, unpredictability, and even desire for social distance.^[21]^ This stigmatization may be a barrier for patients with mental disorder to receive the treatment they need.^[22]^

Pain perception involves peripheral afferent pathways and CNS processing of nociceptive signals. The mechanisms underlying the insensitivity to pain in schizophrenia or dementia are largely unknown, although some notions have been postulated.^[15,23]^ For example, dysfunctions of opioid receptor- and N-methyl-D-aspartate receptor-mediated neurotransmission in CNS have been suggested to participate in the increase in pain threshold in patients with schizophrenia.^[15]^ Additionally, age-related loss in the structure and function of the peripheral and CNS pathways has been implicated in the reduced pain sensitivity in patients with dementia.^[23]^ Furthermore, the inability to successfully communicate pain in these patients may be a contributing factor.^[23]^

The present study had some limitations that should be addressed. Some information that could be relevant, such as time of admission after the development of symptoms, lifestyle of the subjects, patient's clinical presentation, vital signs, laboratory test results, and imaging study reports could not be accessed using the claims-based data, and, therefore, could not be controlled or adjusted. Additionally, we were also unable to interview patients to confirm the diagnosis and obtain information regarding their pain perception. Further studies overcoming these issues are required.

In summary, this population-based case-control study identifies schizophrenia and dementia as risk factors for perforated appendicitis in patients with acute appendicitis. This association may be due to the possibility that patients with psychotic or mental disorders are relatively pain insensitive, resulting in difficulties in the diagnosis and surgical treatment of acute appendicitis. The accuracy of prompt diagnosis of appendicitis remains challenging in such specific populations. Future prospective large-scale studies are still necessary to explore and confirm this clinical issue. Further, studies are also needed to determine the factors that affect pain insensitivities and to understand the underlying mechanisms.

## Author contributions

**Data curation:** Hsiang Chi Wang, Jen Hung Wang.

**Formal analysis:** Hsiang Chi Wang, Jen Hung Wang.

**Investigation:** Huang Ren Lin, Hsin Han Lu.

**Methodology:** Hsiang Chi Wang, Jen Hung Wang.

**Supervision:** Jen Hung Wang.

**Validation:** Jen Hung Wang.

**Writing – original draft:** Huang Re Lin, Hsin Han Lu.

**Writing – review & editing:** Huang Ren Lin, Hsin Han Lu.
